# Effectiveness of Artificial Intelligence Systems in Healthcare: A Systematic Review of Clinical and Organizational Outcomes

**DOI:** 10.3390/healthcare14203532

**Published:** 2026-10-20

**Authors:** Andrea Ceccarelli, Tommaso Montagnani, Tristan Darius Marin, Vincenzo Cassetta, Marco Senni, Marco Montalti, Francesco Sintoni, Davide Gori

**Affiliations:** 1 Department of Medicine and Surgery, University of Milano-Bicocca, Via Cadore 48, 20900 Monza, Italy; 2 Romagna Local Health Authority, Forlì Health District, 47121 Forlì, Italy; 3 Unit of Hygiene and Medical Statistics, Department of Biomedical and Neuromotor Sciences, University of Bologna, 40126 Bologna, Italy; 4 Department of Public Health, Azienda USL di Bologna, Via Castiglione 29, 40124 Bologna, Italy; 5 Nursing Unit, Primary Care and Community Medicine Department of Forlì-Cesena, Romagna Local Health Authority, Via Carlo Boni 67, 47521 Cesena, Italy; 6 Romagna Local Health Authority, Cesena and Rubicone Health District, 47121 Forlì, Italy

**Keywords:** artificial intelligence, healthcare, systematic review, clinical outcomes, organizational outcomes, randomized controlled trial, machine learning, clinical decision support, conversational agents, mHealth, therapeutic adherence

## Abstract

Background: The integration of artificial intelligence (AI)-based systems into healthcare is accelerating rapidly, yet evidence of their real-world clinical and organizational effectiveness remains fragmented. Methods: A PRISMA 2020-compliant systematic review of randomized controlled trials (RCTs) evaluating AI-based interventions in real-world healthcare settings was conducted. MEDLINE (PubMed), CINAHL, Cochrane CENTRAL, and ClinicalTrials.gov were searched up to January 2026. AI interventions were classified post hoc into three functional categories: perceptive AI, predictive/decision support AI, and conversational/adaptive AI. The direction of effect was classified as positive, neutral, mixed, or negative based on the primary endpoint, effect magnitude, and authors’ interpretation. Risk of bias was assessed using the Cochrane RoB-2 tool. Results: A total of 54 RCTs were included. Overall, 40 studies (74.1%) reported a positive direction of effect, nine a neutral result, four a mixed result, and one a negative result. Perceptive AI (*n* = 17) showed consistent benefits in diagnostic accuracy. Predictive/decision support AI (*n* = 15) showed heterogeneous results, with effectiveness dependent on workflow integration. Conversational/adaptive AI (*n* = 22) showed the most consistent positive signal, particularly for chronic disease self-management, behavioral change, and therapeutic adherence. Conclusions: Evidence from randomized trials suggests that AI-based interventions may provide meaningful benefits in real-world healthcare settings, with effectiveness varying by functional category, implementation context, and outcome domain. Future research should prioritize pragmatic multicenter trials with standardized reporting of clinical, organizational, and implementation outcomes.

## 1. Introduction

Artificial intelligence (AI), encompassing machine learning, deep learning, and natural language processing, is increasingly applied across healthcare settings to support clinical decision-making, diagnostic processes, patient communication, and care management [1,2,3]. The rapid expansion of AI-based tools has generated substantial interest in their potential to improve diagnostic accuracy, personalize therapeutic interventions, reduce administrative burdens, and optimize the use of healthcare resources [4,5,6,7]. In parallel, growing evidence from regulatory agencies and international health organizations has underscored the importance of evaluating AI not only for technical performance but also for real-world clinical effectiveness, patient safety, and system-level impact [1,8,9].

Despite this rapidly expanding evidence base, important methodological gaps persist. Most existing evaluations focus on technical validation or single outcome domains, and few systematic reviews have simultaneously assessed both the clinical and organizational consequences of AI implementation in real-world healthcare settings [10]. Furthermore, the heterogeneity of AI technologies, ranging from image recognition systems and clinical decision support tools to conversational agents and adaptive behavioral interventions, makes cross-study synthesis challenging in the absence of a principled classification framework. The current evidence base would benefit from a synthesis organized according to the functional role of the AI intervention rather than the underlying computational architecture, consistent with the perspectives proposed by international frameworks including the WHO, OECD, FDA, and the European Commission [1,11,12,13,14].

Several systematic reviews and scoping reviews have previously examined AI-based interventions in clinical settings. Han et al. identified 86 RCTs evaluating AI in clinical practice up to 2023, finding that the majority reported improvements in clinical outcomes [15]. Liu et al. similarly reviewed AI-assisted tools in clinical care through 2021, noting that most studies reported benefits for AI-assisted clinicians, although single-center designs and limited sample sizes constrained generalizability [16]. The present review builds on these contributions by extending the evidence window to January 2026, by introducing a functional classification of AI interventions grounded in international regulatory frameworks, and by explicitly including organizational alongside clinical outcomes as synthesis targets. The present study aims to address these gaps through a systematic review exclusively of randomized controlled trials (RCTs), evaluating the effectiveness of AI-based systems across both clinical and organizational outcome domains in real-world healthcare settings. The central research question is the following: what is the effectiveness, both clinical and organizational, of the introduction, implementation, and use of AI-based systems in healthcare?

## 2. Materials and Methods

This systematic review was conducted and is reported in accordance with the Preferred Reporting Items for Systematic Reviews and Meta-Analyses (PRISMA) 2020 guidelines [17] and is registered on PROSPERO (CRD420251036790).

### 2.1. Eligibility Criteria

Studies were included if they (1) were randomized controlled trials (RCTs) of any design (parallel-group, crossover, cluster, or stepped-wedge); (2) evaluated AI-based interventions in real-world healthcare settings; (3) reported at least one clinical outcome (e.g., diagnostic accuracy, treatment success, symptom reduction, mortality) and/or organizational outcome (e.g., workflow efficiency, decision time, resource utilization, cost); and (4) were published in English between January 2015 and April 2025. Studies were excluded if they were systematic reviews, meta-analyses, scoping reviews, or study protocols without results or if they were conducted exclusively in laboratory or radiological settings without a real-world clinical component. For the purposes of this review, an AI-based intervention was operationally defined as a system employing at least one of the following technical approaches: machine learning (including deep learning and reinforcement learning), natural language processing, computer vision, or adaptive algorithms that modify their output based on incoming data without explicit reprogramming. Systems based exclusively on pre-specified, static rule sets or conventional branching logic were not considered AI-based and were excluded. The AI component was required to be prospectively and actively deployed as the primary or co-primary intervention within the randomized comparison—that is, the randomized contrast had to directly estimate the effect of the AI system on the outcome of interest. Studies that used RCT-derived data exclusively for retrospective model development or post hoc predictive modeling, without the AI system itself constituting the randomized intervention, were excluded. The real-world healthcare setting criterion required that interventions be implemented with actual patients in routine or near-routine clinical, community, or home-based care contexts; studies conducted exclusively in laboratory settings, on healthy volunteers without a clinical condition, or using simulated or mock consultations were not eligible.

### 2.2. Search Strategy

To address the primary research objective, a PICO framework was formulated as follows: (P) patients or professionals in real-world healthcare settings; (I) implementation or use of AI-based systems or software; (C) standard of care or non-AI approaches; (O) clinical outcomes (e.g., diagnostic accuracy, treatment success, symptom reduction, mortality) and/or organizational outcomes (e.g., workflow efficiency, decision time, resource utilization, cost-effectiveness).

This systematic review was conducted in accordance with the PRISMA 2020 guidelines [17] and registered on PROSPERO (registration number CRD420251036790; registered 2025; available at https://www.crd.york.ac.uk/PROSPERO/view/CRD420251036790, accessed on 12 October 2026). The following deviations from the registered protocol were implemented and are disclosed here: (1) the Cochrane Central Register of Controlled Trials (CENTRAL) was added to the search strategy following reviewer recommendations, as it was not included in the original registration; (2) the search window was extended from April 2025 to January 2026 to capture the most current evidence; (3) two studies (Kim et al. and Lenhard et al.) initially identified as potentially eligible were excluded following re-adjudication against the operationalized AI eligibility criteria (see Section 2.1), as the AI model was not the prospectively deployed randomized intervention in either case. These deviations were imposed to strengthen the methodological rigor of the review and do not affect the core research question or the registered primary outcomes [18,19]. A systematic literature search was conducted across MEDLINE (PubMed), CINAHL (EBSCO), ClinicalTrials.gov, and the Cochrane Central Register of Controlled Trials (CENTRAL) up to January 2026. The search aimed to identify all published articles evaluating the effectiveness of AI-based systems in healthcare, with a focus on studies published within the period January 2015–January 2026 to capture relevant advancements across the past decade. The inclusion criteria were restricted to studies conducted in human participants, including randomized controlled trials (RCTs) of any design (parallel-group, crossover, cluster, or stepped-wedge), with full-text availability in English. Search strategies were tailored to the requirements of each database. Finally, a snowball technique was applied by examining the reference lists of all included primary papers to identify additional eligible studies. Further details of the search strings used across databases are provided in the Supplementary Materials.

### 2.3. Screening and Study Selection Process

**Figure 1:**
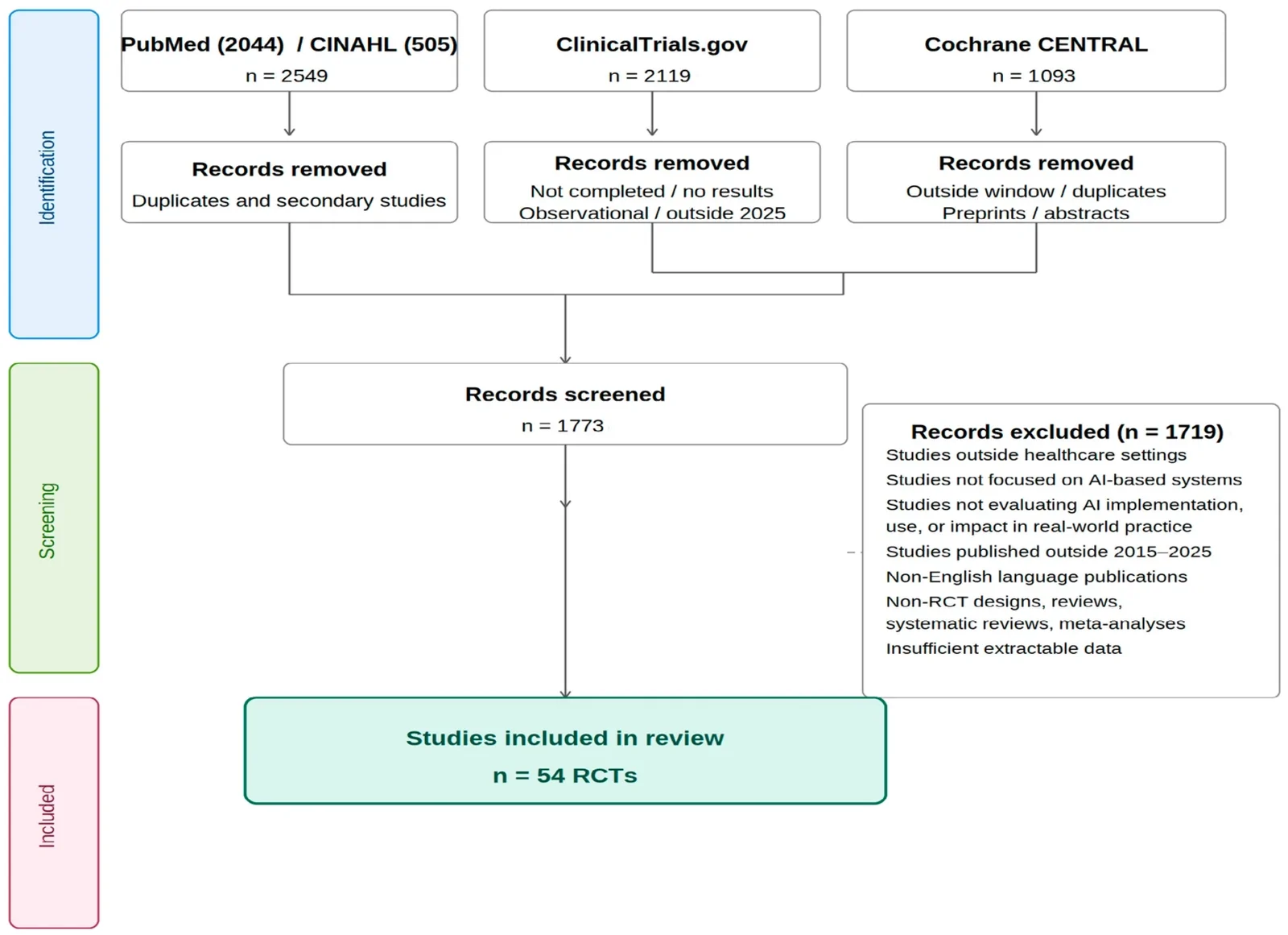
PRISMA 2020 flow diagram of study selection. Records were identified from PubMed/CINAHL (*n* = 2549), ClinicalTrials.gov (*n* = 2119), and Cochrane CENTRAL (*n* = 1093). After the removal of duplicates and non-eligible records, sequential screening was performed. A total of 54 RCTs were included in the final systematic review. Colors indicate the PRISMA 2020 screening phases: blue = Identification; green = Screening; teal = Included.

Titles and abstracts were screened independently by two reviewers against the inclusion and exclusion criteria, followed by the full-text assessment of potentially eligible records. Records retrieved from ClinicalTrials.gov were handled as trial registrations: for each registration, associated results publications were identified via the linked results section and cross-referenced with PubMed and CINAHL to avoid duplication; only registrations with posted results within the eligibility window were carried forward to full-text screening. Disagreements at each stage were resolved by discussion or, when necessary, arbitration by a third reviewer. Of the 1773 records screened, 1719 were excluded based on the pre-specified criteria; the remaining 54 records were assessed in full and all were included in the final review. The complete selection process and reasons for exclusion are reported in the PRISMA 2020 flow diagram (Figure 1). In cases where multiple publications referred to the same trial (e.g., interim reports and final results), only the publication reporting the most complete or longest-term data was included; earlier publications of the same trial were excluded to avoid duplicate reporting of the same study population.

### 2.4. Data Extraction

Data were extracted independently by two reviewers using a standardized form. For each included study, the following variables were recorded: author and year, country, study design, population characteristics (N, key features), type of AI technology, AI function/target, comparator target, primary outcome, key result (quantitative), direction of effect, and outcome domain.

AI interventions were classified post hoc into three functional categories. The classification was based on the predominant clinical function of the AI system, rather than on the underlying computational algorithm, and was performed independently by two reviewers using a pre-defined classification guide grounded in established international frameworks. Initial inter-reviewer agreement on functional category assignment was 90.7% (49 of 54 studies); the five discordant cases were resolved by consensus discussion involving a third reviewer, consistent with published standards for post hoc taxonomy development in systematic reviews. Consistent with the functional perspectives proposed by the WHO [1], OECD [11], FDA [12], and European Commission [13], and aligned with the synthesis framework of Martínez-Millana et al. [14], interventions were categorized into three groups: (i) perceptive AI, encompassing systems that recognize, detect, classify, or quantify clinically relevant patterns from complex unstructured data (medical images, physiological signals, video streams, or sensor-derived data); (ii) predictive/decision support AI, including models that estimate future outcomes, stratify patient risk, or generate recommendations to support clinical decision-making; (iii) conversational/adaptive AI, comprising systems that interact directly with patients or healthcare professionals through natural language or adaptive digital interfaces to deliver personalized education, coaching, monitoring, or behavioral support.

Studies were classified according to the primary clinical purpose of the AI intervention rather than the underlying computational algorithm. This functional classification was developed by integrating the principal application domains consistently identified across international AI frameworks and recent reviews on AI in healthcare [1,6,10,11,12,14]. Specifically, the AI function/target for each included study was assigned to one of five pre-defined categories based on the predominant clinical function of the AI system.

**Diagnosis:** AI systems that detect, classify, or recognize diseases or clinically relevant abnormalities from patient data (e.g., CADe systems for colonoscopy, ECG-based arrhythmia detection, symptom checkers).

**Treatment/Monitoring:** AI systems that directly support treatment delivery, patient self-management, therapeutic adherence, rehabilitation, or longitudinal monitoring of clinical parameters (e.g., adaptive mHealth applications, AI-guided rehabilitation systems, CPAP monitoring).

**Clinical Decision Support (CDSS):** AI systems providing recommendations, alerts, prioritization, or treatment suggestions to healthcare professionals, integrated within clinical workflows or electronic health record systems.

**Prediction:** AI systems estimating future clinical events, disease progression, treatment response, or patient risk based on structured clinical data or multimodal inputs (e.g., prognostic models, ML-based risk stratification).

**Education/Training:** AI systems designed primarily to educate or train patients (e.g., carbohydrate-counting applications for diabetes management) or healthcare professionals (e.g., AI-based surgical simulation tutoring systems).

Outcome domains were classified as clinical (any measure directly related to patient health status, including diagnostic accuracy, symptom reduction, mortality, adherence, and quality of life); organizational (any measure related to healthcare system functioning, professional behavior, or resource utilization, including workflow efficiency, decision time, alert acceptance, and cost); or mixed (both clinical and organizational domains reported within the same study). The direction of effect was categorized as positive (statistically significant improvement in the primary outcome favoring the AI intervention), neutral (no statistically significant difference between groups), mixed (significant improvement in some but not all primary outcomes), or negative (results favoring the control group or standard care).

### 2.5. Risk of Bias Assessment

Risk of bias was assessed independently by two reviewers using the Cochrane Risk of Bias 2 (RoB-2) tool for randomized trials [20]. Each study was evaluated across five domains: (1) bias arising from the randomization process; (2) bias due to deviations from the intended interventions; (3) bias due to missing outcome data; (4) bias in the measurement of the outcome; and (5) bias in the selection of the reported result. For crossover RCTs, the RoB-2 variant for crossover trials was applied, with additional attention to potential carryover effects and the appropriateness of the washout period. For cluster RCTs, the cluster-randomized variant of RoB-2 was used, incorporating domain-specific signaling questions on recruitment bias and the handling of cluster-level confounding. Overall RoB judgements (low risk, some concerns, or high risk) were assigned based on the most critical domain-level concern. Disagreements between reviewers were resolved by discussion or, when necessary, by the arbitration of a third senior reviewer. The potential influence of studies rated as having a high risk of bias on the overall findings is discussed narratively in the Limitations section (Section 4.4). AI-specific reporting and evaluation considerations were informed by the DECIDE-AI framework [21].

### 2.6. Data Synthesis

Given the substantial clinical and methodological heterogeneity across the included studies—spanning diverse AI technologies, hardware configurations, clinical settings, patient populations, comparators, and outcome measures—the meta-analytic pooling of effect estimates was considered inappropriate across all three functional categories. Within the perceptive AI subgroup, although six studies evaluated CADe systems for colonoscopy, with adenoma detection as a common outcome, pooling was precluded by differences in the CADe system architecture, endoscopist experience levels, bowel preparation protocols, and patient eligibility criteria across centers, which represent sources of clinical heterogeneity sufficient to render a pooled estimate uninterpretable. A structured narrative synthesis was therefore performed following the SWiM (Synthesis Without Meta-Analysis) reporting guidance [22], grouping studies according to the three functional AI categories identified post hoc. Within each category, studies were further grouped by AI functional target (diagnosis, treatment/monitoring, CDSS, prediction, education/training) and outcome domain (clinical, organizational, mixed). The primary outcome hierarchy for each included study was pre-specified by the original trial authors and reported as such; the direction of effect was classified as positive, neutral, mixed, or negative based on the trial’s own primary endpoint and its statistical significance, the effect magnitude, and the authors’ own interpretation—not solely on the basis of *p*-value thresholds. For each functional category, the synthesis addressed the number of included studies, direction of effect distribution, consistency of findings across studies, overall methodological quality (RoB profile), and key moderating factors identified across studies. Where individual study effect estimates were available, these are reported with their 95% confidence intervals in the narrative and in Table 1.

## 3. Results

### 3.1. Study Selection

The updated database searches retrieved 2549 records from PubMed and CINAHL combined, 2119 records from ClinicalTrials.gov, and 1093 records from Cochrane CENTRAL (total = 5761). After the removal of duplicates, secondary studies, records outside the eligibility window, preprints, and conference abstracts, a total of 1773 records were screened. Of these, 1719 were excluded based on the pre-specified eligibility criteria (studies outside healthcare settings, not focused on AI-based systems, not evaluating AI implementation in real-world practice, published outside the 2015–2025 window, non-English language, non-RCT designs including reviews and meta-analyses, and insufficient extractable data). A final total of 54 RCTs were included in the systematic review. The complete selection process is illustrated in Figure 1 (PRISMA 2020 flow diagram).

The complete characteristics of all 54 included studies, including the AI technology type, AI functional target, primary outcome, key results, direction of effect, and outcome domain, are presented in Table 1 below.

### 3.2. Characteristics of Included Studies

A total of 54 RCTs were included in the systematic review, published between 2017 and 2025. Studies were conducted across 17 countries, with the USA (*n* = 17), China (*n* = 8), Republic of Korea (*n* = 5), Spain (*n* = 4), and Canada (*n* = 4) being the most represented. The remaining studies originated from Australia, Denmark, Egypt, the UK, Ireland, Italy, Japan, The Netherlands, Sweden, Taiwan, and multinational settings. Sample sizes ranged from 20 (Kanazawa et al. [39]) to 288,832 clinical interactions (Wright et al. [71]). The large majority were parallel-group RCTs (*n* = 36). Additional designs included crossover RCTs (*n* = 4), cluster RCTs (*n* = 4), pilot RCTs (*n* = 2), open-label RCTs (*n* = 3), and one tandem RCT. Clinical populations varied widely, encompassing patients with chronic non-communicable diseases (diabetes, heart failure, chronic pain, COPD, obstructive sleep apnea), mental health conditions (depression, anxiety, OCD, schizophrenia, substance misuse, eating disorders), and cancer; surgical or procedural contexts; and healthy adults or healthcare professionals. The full characteristics of all included studies are reported in Table 1. The included studies exhibited substantial heterogeneity across multiple dimensions, which precluded meta-analytic pooling and informed the choice of a structured narrative synthesis. Regarding the AI technology, 54 distinct systems were evaluated, ranging from deep learning computer vision models for endoscopic detection to reinforcement learning chatbots for smoking cessation, with no two studies evaluating the same AI system in the same clinical context. Comparators varied from no intervention or waiting list (*n* = 12) to standard of care (*n* = 30) to active human-delivered equivalents such as telephone-based CBT or nurse-led programs (*n* = 12). The follow-up duration ranged from 2 weeks (Fitzpatrick et al. [33], Ly et al. [51]) to 48 weeks (Lee et al. [46]) and up to 12 months (Piette et al. [58], Carrasco-Hernández et al. [28]). Primary outcomes spanned at least 30 distinct measurement instruments, including the adenoma detection rate, HbA1c, PHQ-9, Roland-Morris Disability Questionnaire, CPAP adherence, alert acceptance rate, and all-cause mortality, making a direct cross-study comparison of effect sizes methodologically inappropriate. Sample sizes varied by more than three orders of magnitude, from *n* = 20 (Kanazawa et al. [43]) to *n* = 288,832 clinical interactions (Wright et al. [74]).

### 3.3. AI Functional Taxonomy

Based on the post hoc classification, AI interventions were distributed across three technology types: perceptive AI (*n* = 17), predictive/decision support AI (*n* = 15), and conversational/adaptive AI (*n* = 22). The AI functional targets were as follows: diagnosis (*n* = 12), treatment/monitoring (*n* = 24), clinical decision support/CDSS (*n* = 10), prediction (*n* = 4), and education/training (*n* = 4). Regarding the outcome domain, 34 studies reported exclusively clinical outcomes, three reported exclusively organizational outcomes, and 17 reported mixed outcomes spanning both clinical and organizational domains. As shown in Table 2, the distribution of outcome domains differed substantially across AI functional categories. Perceptive AI studies focused almost exclusively on clinical outcomes (14 of 17; 82.4%), consistent with their predominant application in diagnostic and image analysis tasks, where clinical endpoints are the natural primary measure. Predictive/decision support AI showed the highest proportion of organizational outcomes (2 of 15; 13.3%) and mixed outcomes (4 of 15; 26.7%), reflecting the dual focus of CDSS applications on both patient health and clinician workflows. Conversational/adaptive AI had the largest proportion of mixed outcomes (10 of 22; 45.5%), driven by studies that measured both patient-level behavioral change and implementation-level indicators such as engagement rates, adherence, and therapist time savings. Purely organizational outcomes were rare across all three categories (*n* = 3 total).

### 3.4. Risk of Bias

Of the 54 included RCTs, 16 were rated as having a low risk of bias [27,32,36,37,43,44,48,52,60,63,68,70,71,72,73,74], 26 as having some concerns [24,25,26,30,31,34,38,39,41,42,46,47,49,50,53,55,57,58,61,62,64,65,66,67,69,76], and 12 as having a high risk [23,28,29,33,35,40,45,51,54,56,59,75] (Table 3). Table 3 provides the complete RoB-2 profile for each included study and the color-coded overall judgment, which complements the summary counts reported in this section and is intended as a reference tool for readers wishing to assess the methodological profiles of individual studies. Studies rated as low-risk generally demonstrated adequate allocation concealment, pre-registered primary outcomes, and objective or blinded outcome assessment. The most common source of risk in the some concerns category was the potential for deviations from the intended interventions, particularly in open-label designs, where the blinding of participants to AI-based interventions was inherently unfeasible. Studies rated as high-risk were predominantly characterized by the absence of allocation concealment, high dropout rates, self-reported outcomes without objective validation, or a lack of pre-registration of primary endpoints.

**Table 3:** Risk of bias assessment of included RCTs using the Cochrane Risk of Bias 2 (RoB-2) tool. Color coding: green = low risk; yellow = some concerns; red = high risk.

Authors	Tool for Assessment	Risk of Bias
Aguilera et al., 2024 [23]	Cochrane RoB-2	High
Ahmad et al., 2023 [24]	Cochrane RoB-2	Some Concerns
Alfonsi et al., 2020 [25]	Cochrane RoB-2	Some Concerns
Anan et al., 2021 [26]	Cochrane RoB-2	Some Concerns
Blomberg et al., 2021 [27]	Cochrane RoB-2	Low
Carrasco-Hernandez et al., 2020 [28]	Cochrane RoB-2	High
Dean et al., 2022 [29]	Cochrane RoB-2	High
Desai et al., 2023 [30]	Cochrane RoB-2	Some Concerns
Doherty et al., 2024 [31]	Cochrane RoB-2	Some Concerns
Fazlollahi et al., 2022 [32]	Cochrane RoB-2	Low
Fitzpatrick et al., 2017 [33]	Cochrane RoB-2	High
Fritz et al., 2024 [34]	Cochrane RoB-2	Some Concerns
Fulmer et al., 2018 [35]	Cochrane RoB-2	High
Gong et al., 2020 [36]	Cochrane RoB-2	Low
Halcox et al., 2016 [37]	Cochrane RoB-2	Low
Harry et al., 2022 [38]	Cochrane RoB-2	Some Concerns
He et al., 2024 [39]	Cochrane RoB-2	Some Concerns
Huan Hwa Chen et al., 2023 [40]	Cochrane RoB-2	High
Itoh et al., 2022 [41]	Cochrane RoB-2	Some Concerns
Jayakumar et al., 2021 [42]	Cochrane RoB-2	Some Concerns
Kanazawa et al., 2023 [43]	Cochrane RoB-2	Low
Knitza et al., 2024 [44]	Cochrane RoB-2	Low
Lee, Byun et al., 2025 [45]	Cochrane RoB-2	High
Lee et al., 2023 [46]	Cochrane RoB-2	Some Concerns
Li et al., 2025 [47]	Cochrane RoB-2	Some Concerns
Li et al., 2024 [48]	Cochrane RoB-2	Low
Lin et al., 2024 [49]	Cochrane RoB-2	Some Concerns
Lustrek et al., 2021 [50]	Cochrane RoB-2	Some Concerns
Ly et al., 2017 [51]	Cochrane RoB-2	High
Manz et al., 2023 [52]	Cochrane RoB-2	Low
Mathioudakis et al., 2025 [53]	Cochrane RoB-2	Some Concerns
Nayak et al., 2023 [54]	Cochrane RoB-2	High
Nicolae et al., 2020 [55]	Cochrane RoB-2	Some Concerns
Olano-Espinosa et al., 2022 [56]	Cochrane RoB-2	High
Pelle et al., 2020 [57]	Cochrane RoB-2	Some Concerns
Piette et al., 2022 [58]	Cochrane RoB-2	Some Concerns
Prochaska et al., 2021 [59]	Cochrane RoB-2	High
Repici et al., 2020 [60]	Cochrane RoB-2	Low
Sadeh-Sharvit et al., 2023 [61]	Cochrane RoB-2	Some Concerns
Seol et al., 2021 [62]	Cochrane RoB-2	Some Concerns
Shade et al., 2025 [63]	Cochrane RoB-2	Low
Sharma et al., 2023 [64]	Cochrane RoB-2	Some Concerns
Sharma et al., 2025 [65]	Cochrane RoB-2	Some Concerns
Sharp et al., 2025 [66]	Cochrane RoB-2	Some Concerns
Sibley et al., 2025 [67]	Cochrane RoB-2	Some Concerns
Su et al., 2020 [68]	Cochrane RoB-2	Low
Tawfik et al., 2023 [69]	Cochrane RoB-2	Some Concerns
Turino et al., 2021 [70]	Cochrane RoB-2	Low
Wallace et al., 2022 [71]	Cochrane RoB-2	Low
Wang et al., 2020 [72]	Cochrane RoB-2	Low
Wijnberge et al., 2020 [73]	Cochrane RoB-2	Low
Wright et al., 2023 [74]	Cochrane RoB-2	Low
Xiao et al., 2025 [75]	Cochrane RoB-2	High
Yao et al., 2021 [76]	Cochrane RoB-2	Some Concerns

### 3.5. Narrative Synthesis by AI Functional Category

Given the clinical and methodological heterogeneity across the included studies, a structured narrative synthesis was conducted, grouping studies by the three functional AI categories identified post hoc. For each category, the synthesis addresses the number of studies, direction of effect, consistency of results, overall methodological quality, and key moderating factors.

#### 3.5.1. Perceptive AI

Seventeen studies evaluated perceptive AI systems—technologies that recognize clinically relevant patterns from complex unstructured data, including endoscopic video, physiological signals, and sensor-based inputs. Eight studies targeted diagnosis, four targeted treatment/monitoring, four targeted education/training, and one targeted prediction.

Diagnostic AI—endoscopy and image-based detection. Six studies evaluated computer-aided detection (CADe) systems for colonoscopy. Repici et al. [60] (Italy, *n* = 685) demonstrated that real-time CADe significantly increased the adenoma detection rate (ADR) from 40.4% to 54.8% (RR 1.30). Gong et al. [36] (China, *n* = 704) reported the doubling of the ADR with the ENDOANGEL system (Wuhan EndoAngel Medical Technology Co., Ltd., Wuhan, China) (16% vs. 8%, *p* = 0.001). Su et al. [68] (China, *n* = 623) showed an ADR improvement from 16.5% to 28.9% alongside significant reductions in polyp miss rates. Ahmad et al. [24] (UK, *n* = 614) demonstrated a significant polyp detection rate improvement (79.2% vs. 71.6%, *p* = 0.03), but the primary ADR endpoint narrowly missed significance (*p* = 0.09), yielding a mixed result. Wang et al. [72] (China, *n* = 962) reported significant reductions in adenoma miss rates (13.9% vs. 40.0%), also classified as mixed due to variability across colonic segments. Wallace et al. [71] (Italy/UK/USA, *n* = 230) evaluated AI-assisted tandem colonoscopy in a multicenter setting, demonstrating a significantly lower adenoma miss rate in the AI-first arm (15.5% vs. 32.4%, adjusted OR 0.38 [95% CI 0.23–0.62]) and a substantially lower false-negative rate (6.8% vs. 29.6%), supporting a positive direction of effect. Desai et al. [30] (USA, *n* = 1033) reported significantly more adenomas per colonoscopy in the AI-assisted arm (0.99 vs. 0.85, IRR 1.17 [95% CI 1.03–1.33], *p* = 0.02), also classified as positive.

**Diagnostic AI—cardiovascular and emergency settings.** Halcox et al. [37] (UK, *n* = 1000) suggested that an AI-based handheld ECG device (AliveCor, AliveCor, Inc., Mountain View, CA, USA) significantly increased new atrial fibrillation diagnoses versus routine care (HR = 3.9, 95% CI 1.4–10.4, *p* = 0.007). Blomberg et al. [27] (Denmark, *n* = 5242 calls) found that a machine learning system identified out-of-hospital cardiac arrest with 85% sensitivity; however, the dispatcher recognition rate did not improve significantly (93.7% vs. 90.5%, *p* = 0.15) because operators frequently disregarded the alerts, yielding a neutral direction of effect on the primary outcome.

Perceptive AI in treatment/monitoring and education/training. He et al. [39] (China, *n* = 75) evaluated a 3D pose estimation system for remote rehabilitation in older adults with sarcopenia; AI-based tele-training was non-inferior to in-person training across all functional outcomes but did not demonstrate superiority (neutral). Huan Hwa Chen et al. [40] (Taiwan, *n* = 94) reported that an AI-assisted medication adherence monitoring system for patients with schizophrenia significantly improved adherence (94.7% vs. 64.4%, *p* < 0.001), with concurrent improvements in symptom severity and cognition (positive). Xiao et al. [75] (China, *n* = 38) demonstrated significantly greater reductions in pain with AI-guided home telerehabilitation for chronic low back pain (most severe NRS: −3.00 vs. −1.50, adjusted mean difference −1.08 [95% CI −1.68 to −0.49], *p* < 0.001) versus video-based exercise (positive). Sharma et al. [65] (UK, *n* = 396) evaluated an AI-assisted tele-ophthalmology pathway for glaucoma referrals, reporting a 59% reduction in false-positive urgent referrals versus standard care, although the AI model’s sensitivity and specificity were lower than those of human experts (mixed). Alfonsi et al. [25] (Canada, *n* = 44) demonstrated that an AI-based carbohydrate-counting application significantly improved accuracy (*p* = 0.008), reduced large errors (*p* = 0.047), and lowered HbA1c (*p* = 0.03) in youth with type 1 diabetes (positive).

Overall, 11 of 17 perceptive AI studies reported a positive direction of effect, three reported mixed findings (Ahmad et al. [24], Wang et al. [72], Sharma et al. [65]), two were neutral (Blomberg et al. [27], He et al. [39]), and one was positive with caveats (Xiao et al. [75]). The mixed results in the CADe colonoscopy subgroup reflected cases where the primary ADR endpoint narrowly missed statistical significance despite significant improvements in secondary detection metrics. The neutral result in the cardiac arrest detection study illustrates the recurrent pattern of high technical performance without a corresponding clinical impact, attributable to the low uptake of AI alerts by human operators.

#### 3.5.2. Predictive/Decision Support AI

Fifteen studies evaluated predictive/decision support AI systems. Ten were classified as CDSS, three as prediction, one as education/training, and one as diagnosis.

Clinical Decision Support Systems (CDSS). The results were heterogeneous, with a clear distinction between studies where AI was structurally embedded into clinical workflows and those delivering passive alerts. Dean et al. [29] (USA, *n* = 6848) reported a 38% reduction in 30-day all-cause mortality in pneumonia patients (aOR 0.62, *p* < 0.001), alongside a 17% increase in direct home discharge. Manz et al. [52] (USA, *n* = 20,506) reported a sustained increase in documented serious illness conversations from 3.4% to 13.5% (aOR 2.62, *p* < 0.001) when using machine learning-triggered behavioral nudges. Sadeh-Sharvit et al. [61] (USA, *n* = 47) showed that an AI behavioral intervention platform reduced therapist administrative time from 69 to 14 h (*p* < 0.001) without compromising clinical outcomes. Kanazawa et al. [43] (Japan, *n* = 20) demonstrated a significant improvement in resident physicians’ diagnostic accuracy (56.1% vs. 39.3%, *p* = 0.02). Nicolae et al. [55] (Canada, *n* = 41) demonstrated the noninferiority of AI-based brachytherapy planning with significantly reduced planning time. Jayakumar et al. [42] (USA, *n* = 129) evaluated an AI-enabled patient decision aid for knee arthroplasty, demonstrating significantly higher decision quality (K-DQI mean difference 20.0%, 95% CI 14.2–26.1%, *p* < 0.001), better shared decision-making, and improved functional outcomes at 4–6 months. Sibley et al. [67] (USA, *n* = 51 adolescents with ADHD) evaluated an AI-assisted fidelity support system for community-based therapy, finding more efficient treatment delivery but no significant improvement in MI quality over time and fewer appointments attended, yielding a mixed result. Regarding the alert fatigue pattern, three studies showed a limited clinical impact attributable to low clinician uptake of AI alerts. Harry et al. [38] (USA, *n* = 5054) found no significant difference in HPV vaccination completion (2.3% vs. 2.2%, *p* = 0.827). Wright et al. [74] (USA, *n* = 288,832) showed that the AI system identified 4.6 times more missing diagnoses (*p* < 0.001) but overall alert acceptance remained at 22.1%, with no significant improvement in downstream quality measures. Seol et al. [62] (USA, *n* = 184) reported mixed results: a dramatic reduction in physician chart review time (3.5 vs. 11.3 min, *p* < 0.001) without a significant change in asthma exacerbations. Fritz et al. [34] (USA, *n* = 5071 surgical patients; 89 clinicians) found no significant improvement in clinician prediction of postoperative complications when supported by machine learning (AUROC mortality: 0.793 vs. 0.780, *p* = 0.76; AUROC AKI: 0.734 vs. 0.688, *p* = 0.06), consistent with the alert fatigue pattern and suggesting that presenting AI-generated risk scores to clinicians as additional information, without workflow integration, may not yield improved decision-making.

Beyond the predominant CDSS applications, the remaining studies addressed prediction, education/training, treatment/monitoring, and diagnosis targets. Wijnberge et al. [73] (The Netherlands, *n* = 68) demonstrated dramatic reductions in intraoperative hypotension using a machine learning prediction index (time-weighted average 0.10 vs. 0.44 mmHg, *p* = 0.001). Itoh et al. [41] (Japan, *n* = 101 workers with chronic low back pain) evaluated an AI-based digital therapeutic for work productivity, finding no significant improvement in the primary endpoint (work productivity difference, QQ: −0.053, *p* = 0.43), yielding a neutral direction of effect. Fazlollahi et al. [32] (Canada, *n* = 70) demonstrated that AI-based surgical tutoring significantly outperformed remote expert instruction (expertise score improvement +0.66 points, *p* < 0.001). Knitza et al. [44] was the only study in the review that reported a negative direction of effect, with limited diagnostic accuracy shown for two AI-based triage tools for inflammatory rheumatic diseases (concordance 58% and 52% with physician diagnosis).

Overall, 11 of 15 predictive/decision support AI studies reported a positive direction of effect (73.3%), while three showed neutral findings, one was mixed, and one was negative. The studies with neutral or limited results shared a common structural feature: AI outputs delivered as an interruptive alert or additional risk score requiring active clinician response, rather than being integrated into the clinical workflow.

#### 3.5.3. Conversational/Adaptive AI

Twenty-two studies evaluated conversational/adaptive AI systems, making this the largest subgroup in the systematic review. All 22 were classified under treatment/monitoring (*n* = 18) or education/training (*n* = 4), reflecting the predominant role of these systems in supporting patient self-management, therapeutic adherence, behavioral change, rehabilitation, monitoring, and patient education across diverse chronic and non-chronic conditions. Nineteen of the 22 studies reported a positive direction of effect; two reported neutral results (Pelle et al. [57], Mathioudakis et al. [53]) and one reported mixed results (Sibley et al. [67], which is included in Section 3.5.2 as predictive/CDSS). The one neutral result within this subgroup that was attributable solely to conversational/adaptive AI was that of Pelle et al. [57] and Mathioudakis et al. [53].

Mental health and substance use. Five studies evaluated conversational AI for psychological or behavioral outcomes. Fitzpatrick et al. [33] (USA, *n* = 70) demonstrated significant reductions in depression (PHQ-9, *p* = 0.01) and anxiety (*p* = 0.004) when using Woebot (Woebot Health, Inc., San Francisco, CA, USA) over two weeks. Fulmer et al. [35] (USA, *n* = 75) reported significant improvements in depression (PHQ-9, *p* = 0.02) and anxiety (GAD-7, *p* = 0.02–0.045) when using the Tess conversational agent (X2AI, Inc., San Francisco, CA, USA). Ly et al. [51] (Sweden, *n* = 28) found improved psychological well-being and reduced stress with the Shim chatbot (Linköping University, Linköping, Sweden) in a pilot study. Prochaska et al. [59] (USA, *n* = 180) reported a significant reduction in substance use occasions (9.1 vs. 3.3 per month, *p* = 0.039) when using Woebot-SUDs. Sharp et al. [66] (Australia, *n* = 60) evaluated a chatbot single-session intervention for patients on a waitlist for eating disorder treatment, demonstrating a significant group × time effect on global EDE-Q scores (F2,116 = 5.98, *p* = 0.003, ηp^2^ = 0.09) and significantly higher rates of treatment initiation (93% vs. 70%, *p* = 0.042). All five studies reported positive effects, although the sample sizes were small and follow-up periods were brief.

Smoking cessation. Two RCTs evaluated AI-based chatbot interventions for smoking cessation, both conducted in Spain. Carrasco-Hernandez et al. [28] (*n* = 240, specialized unit) reported abstinence rates of 64.7% vs. 40.0% per-protocol at one year (intention-to-treat: 27.5% vs. 15.0%, *p* ≤ 0.02). Olano-Espinosa et al. [56] (*n* = 513, primary care) reported a more modest but statistically significant benefit at six months (26.0% vs. 18.8%, *p* = 0.05), confirmed by CO oximetry. Both studies were positive, although the difference in effect size suggests that the clinical context and patient selection may moderate effectiveness.

Chronic disease management and therapeutic adherence. Eight studies addressed the management of chronic conditions. Turino et al. [70] (Spain, *n* = 60) demonstrated a clinically meaningful increase in CPAP adherence (+1.14 h/day, *p* = 0.047) without an additional cost. Nayak et al. [54] (USA, *n* = 32) showed that AI-guided insulin titration achieved optimal glycemic control in 15 days, versus more than 56 days in standard care (81.3% vs. 25.0% reaching target). Aguilera et al. [23] (USA, *n* = 168) demonstrated that adaptive messaging significantly increased daily step counts (+19% in the adaptive arm vs. +3.9% in the control arm). Lustrek et al. [50] (Belgium/Italy, *n* = 56) reported improvements in self-care and reductions in depression and anxiety in stable heart failure outpatients. Lee et al. [46] (Republic of Korea, *n* = 294) evaluated an integrated digital health platform for type 2 diabetes management across three university hospitals, reporting significantly lower HbA1c in both intervention arms versus routine care at 48 weeks (positive). Mathioudakis et al. [53] (USA, *n* = 368) evaluated an AI-powered lifestyle intervention versus human coaching in the Diabetes Prevention Program in a pragmatic noninferiority RCT, demonstrating the noninferiority of AI versus human coaching on the composite primary endpoint (31.7% vs. 31.9%), yielding a neutral result while supporting the scalability of AI-delivered behavioral counseling. Sharma et al. [64] (Canada, *n* = 52) evaluated a voice-assisted AI for SARS-CoV-2 screening in a heart failure clinic, reporting high concordance with healthcare staff (96.9–98.5%, κ 0.92–0.95) but with no significant difference between groups, yielding a neutral result. Pelle et al. [57] (The Netherlands, *n* = 427) reported no significant reduction in secondary healthcare consultations with the Dr. Bart osteoarthritis app (IRR 1.20, *p* > 0.05), although small symptomatic improvements were observed (neutral).

Musculoskeletal conditions, rehabilitation, and behavioral engagement. Five studies addressed physical activity, rehabilitation, or musculoskeletal conditions. Anan et al. [26] (Japan, *n* = 121) demonstrated dramatic improvements in neck, shoulder, and low back pain (OR 43.00, *p* < 0.001; adherence 92%) with a LINE-based chatbot in a workplace setting. Piette et al. [58] (USA, *n* = 439) demonstrated that AI-supported CBT for chronic back pain was noninferior to standard telephone-delivered CBT at significantly lower therapist time (∼50% reduction), with significant reductions in RMDQ and BPI at 3, 6, and 12 months (*p* < 0.001). Li et al. [47] (China, *n* = 47) reported significant improvements in cardiorespiratory capacity (VO_2_ peak +3.66 vs. +1.09 mL/kg/min, *p* = 0.02) and quality of life (*p* = 0.03) with home-based AI-guided rehabilitation in post-surgery lung cancer patients. Doherty et al. [31] (Ireland, *n* = 62) showed that a reinforcement learning exercise application produced significantly higher user satisfaction (PACES-8: 4.0 vs. 3.73, *p* = 0.02) and exercise intensity (5.82 vs. 5.19, *p* < 0.01) versus a standard app. Shade et al. [63] (USA, *n* = 50) reported that personalized morning routines delivered by an AI voice assistant significantly increased engagement rates in older adults with chronic pain (74% vs. 58%, *p* = 0.002).

Education and training. Two studies targeted patient education in oncology settings. Tawfik et al. [69] (Egypt, *n* = 150 breast cancer patients undergoing chemotherapy) evaluated a nurse-led versus chatbot-delivered empowerment program, demonstrating significant improvements in patient-centered symptom outcomes and self-care behaviors in both intervention arms versus routine care (positive). Lee, Byun et al. [45] (Republic of Korea, *n* = 145 breast cancer patients undergoing radiotherapy) evaluated a chatbot and video education intervention for anxiety management in a four-arm RCT, finding no significant group × time interaction for the primary anxiety endpoints, yielding a neutral result across the full sample. Overall, 19 of 22 conversational/adaptive AI studies reported a positive direction of effect. Two studies reported neutral results (Pelle et al. [57] on the primary healthcare utilization endpoint; Mathioudakis et al. [53] on noninferiority to human coaching), and one reported neutral findings, attributable to structural limitations of the delivery modality rather than the inefficacy of AI (Lee, Byun et al. [45]). These considerations are consistent with the methodological profile of the subgroup. Of the 22 included studies, two were rated as having a low risk of bias [63,70], 11 as having some concerns [26,31,46,47,50,53,57,58,64,66,69], and nine as having a high risk [23,28,33,35,45,51,54,56,59]. High-risk ratings were predominantly associated with chatbot studies for mental health and smoking cessation, where blinding was inherently unfeasible, samples were small, follow-up periods were brief, and outcomes were largely self-reported. The positive signal observed across this subgroup should therefore be interpreted cautiously: while the consistency of the findings across diverse clinical conditions and populations is noteworthy, the overall certainty of the evidence remains limited by methodological heterogeneity and the predominance of studies at a high or uncertain risk of bias.

## 4. Discussion

This systematic review synthesized evidence from 54 randomized controlled trials evaluating real-world applications of AI across diverse healthcare domains. The findings indicate a predominantly positive direction of effect (74.1%), although the magnitude and consistency of benefit varied substantially across AI functional categories, implementation contexts, and outcome domains.

### 4.1. From Technical Efficacy to Real-World Effectiveness: The Role of Implementation

One of the most relevant findings of this systematic review is the apparent discrepancy between the technical performance of AI systems and their ability to generate measurable improvements in real-world clinical outcomes. This pattern was particularly evident among predictive/decision support AI interventions. Although 10 of the 14 studies in this category reported a positive direction of effect, the studies classified as neutral or mixed shared an important characteristic: the AI output was frequently delivered as an interruptive alert requiring an active response from clinicians, rather than being seamlessly incorporated into existing clinical workflows. The findings of Wright et al. are illustrative: the AI system identified substantially more potentially missing diagnoses than standard practice, yet overall alert acceptance remained low and no significant improvement in downstream quality measures was observed. A similar pattern was observed by Harry et al., where AI-generated alerts did not significantly increase HPV vaccination completion, and by Seol et al., where a substantial reduction in physician chart review time was not accompanied by a significant reduction in asthma exacerbations. These findings suggest that improved information generation and improved healthcare outcomes should not be considered interchangeable endpoints.

This observation is consistent with broader evidence on the determinants of AI acceptance in clinical settings. Lambert et al. identified difficulties in workflow integration and concerns regarding professional autonomy as important barriers, whereas appropriate training and the early involvement of end users were identified as facilitators [77]. Scott et al. similarly emphasized that large-scale clinician adoption of AI-enabled decision support remains limited despite the rapid proliferation of technically validated systems, highlighting the importance of usability, trust, workflow compatibility, and organizational readiness [78]. The contrast observed within our predictive/decision support category further suggests that AI systems may be more likely to produce meaningful clinical effects when structurally embedded within care processes rather than being added as an additional layer of information: Dean et al. reported a substantial reduction in 30-day mortality in pneumonia patients, and Manz et al. demonstrated a marked and durable increase in serious illness conversations, both in contexts where the AI output was integrated into established clinical workflows [29,52]. The AI for IMPACTS framework, proposed by Jacob et al., formalizes this perspective, arguing that the evaluation of healthcare AI should encompass integration, workflow compatibility, acceptability, training, cost, safety, scalability, and long-term impact as complementary dimensions beyond technical performance alone [79].

It should be noted, however, that conclusions regarding the role of workflow integration are inferential rather than being directly tested within any single included RCT. The distinction between studies where AI was embedded versus added as an alert is an observational pattern emerging from the post hoc comparison across trials rather than a randomized contrast. The follow-up duration in the predictive/CDSS subgroup ranged from a single encounter (Kanazawa et al. [43]) to 12 months (Dean et al. [29]), limiting conclusions about whether early positive effects are sustained. Furthermore, all five studies in which the alert fatigue pattern was most evident were conducted in US academic medical centers, raising the question of whether the pattern would differ in other healthcare systems where the electronic health record infrastructure, clinician workload, and alert cultures differ substantially.

### 4.2. Conversational/Adaptive AI and Patient-Level Behavioral Support

A second major finding concerns the comparatively consistent performance of conversational/adaptive AI, the largest subgroup (16 of 41 studies), with 15 reporting a positive direction of effect. All studies in this category were classified under treatment/monitoring, reflecting their predominant role in supporting patient self-management, therapeutic adherence, monitoring, rehabilitation, and behavioral change. The pattern across these studies suggests that the potential value of conversational/adaptive AI may lie less in replacing clinical decision-making and more in providing continuous, personalized support between formal healthcare encounters, spanning smoking cessation, CPAP adherence, physical activity, chronic disease self-care, pain management, rehabilitation, and mental health.

The study by Piette et al. provides a particularly relevant illustration [58]. In a randomized comparative effectiveness trial, an AI-supported cognitive behavioral therapy intervention using reinforcement learning was noninferior to standard telephone-delivered CBT while requiring substantially less therapist time, with a greater proportion of patients achieving clinically meaningful improvements in pain-related disability at six months. The intervention did not simply automate therapy: the AI system used repeated patient feedback to adapt the intensity and type of subsequent contact, offering a conceptual model for the positive signal observed across conversational/adaptive AI studies in this systematic review.

The broader literature supports this signal while counselling caution regarding heterogeneity. Li et al. found that AI-driven conversational agent interventions showed evidence of a benefit across physical functioning, lifestyle behaviors, and mental health outcomes, although the effects were heterogeneous and the methodological quality varied substantially [80]. Kurniawan et al. similarly reported the favorable acceptance of AI-powered chatbots for chronic disease self-management, while emphasizing that evidence for clinical efficacy remains constrained by the heterogeneous technologies and incomplete reporting of technical characteristics [81]. These considerations are consistent with the methodological profile of the present subgroup. Of the 16 included conversational/adaptive AI studies, eight were rated as having a high risk of bias, six as having some concerns, and only 2 as having a low risk. The potential value of conversational/adaptive AI may therefore extend beyond information delivery, providing repeated, personalized support that can reinforce health-related behaviors between formal healthcare encounters. Evidence from other preventive care settings supports this interpretation: in an Italian oncology context, in-hospital vaccination counselling embedded within an established care pathway was associated with continued adherence to recommended vaccination schedules (OR 7.05, 95% CI 2.29–21.70) [82]. A recent systematic review and meta-analysis further showed that organizational and technological implementations in general practice, including reminder systems, digital outreach, and structured patient contact, were associated with a significant increase in vaccine uptake among adults (OR = 1.15; 95% CI: 1.03–1.27; *p* < 0.0001) [83]. Although neither intervention was AI-based, the underlying mechanism—personalized, technology-enabled contact integrated within care pathways—is conceptually aligned with the behavioral support model of conversational/adaptive AI, which could represent a scalable extension of this approach for vulnerable or difficult-to-reach populations. International immunization strategies, including the WHO Immunization Agenda 2030, similarly emphasize trust, effective communication, and person-centered approaches as key components of successful vaccination programs [84].

### 4.3. Contextual Effectiveness and the Underrepresentation of Organizational Outcomes

Of the 54 included studies, 34 reported exclusively clinical outcomes, three reported exclusively organizational outcomes, and 17 reported mixed outcomes spanning both domains (Table 2). This distribution reflects a fundamental imbalance in the current evidence base: randomized trials of AI in healthcare have predominantly been designed around patient-level endpoints, and the organizational consequences of AI implementation remain substantially underrepresented.

Among the three studies reporting exclusively organizational outcomes, Fazlollahi et al. [32] demonstrated that AI-based surgical tutoring significantly outperformed remote expert instruction in training efficiency (expertise score +0.66, *p* < 0.001). Wright et al. [74] showed that a CDSS identified 4.6 times more missing diagnoses than standard care, although alert acceptance remained at 22.1%. Sibley et al. [67] reported more efficient treatment delivery but fewer appointments attended and declining implementation fidelity over time in a community mental health setting. Among the 17 mixed-outcome studies, the most consistent organizational signal was the reduction in clinician time: Sadeh-Sharvit et al. [61] reduced the therapist administrative time from 69 to 14 h without compromising clinical outcomes, and Piette et al. [58] achieved comparable clinical outcomes to standard telephone CBT at approximately 50% lower therapist time. Cost data were reported in only two studies (Halcox et al. [37]; Turino et al. [70]), and neither demonstrated a statistically significant cost advantage. Implementation and adoption outcomes (alert acceptance, uptake rates, engagement) were reported in six studies, all showing partial uptake that attenuated the potential clinical impact. These findings suggest that the organizational evidence base is currently insufficient to draw conclusions about the cost-effectiveness, scalability, or system-level impact of AI interventions in healthcare.

The findings of this review are broadly consistent with those of prior syntheses of AI RCTs. Han et al. identified 86 RCTs evaluating AI in clinical practice through 2023, reporting that 73.4% of studies showed improvements for AI-assisted clinicians over unassisted care [15]—a figure that is closely aligned with the 74.1% positive rate observed in the present review, despite the different classification framework and extended evidence window. Similarly, Liu et al. reported that most AI RCTs in clinical care reported benefits, although they noted that single-center designs and limited sample sizes constrained the generalizability [16]. The present review extends and complements these prior syntheses in three respects: first, by introducing a functional AI taxonomy that reveals differential effectiveness patterns across perceptive, predictive, and conversational categories; second, by including organizational outcomes as a co-primary synthesis target, revealing their substantial underrepresentation in the current evidence base; and, third, by updating the evidence window to January 2026, capturing 15 additional RCTs published after the search cutoff of Han et al. The convergence of our positive rate with theirs, despite these methodological extensions, strengthens the confidence in the directional signal, while the taxonomy analysis adds interpretive depth that aggregate positive rate figures alone cannot provide.

The relative scarcity of organizational outcomes may reflect a broader characteristic of the current evidence base: randomized trials of AI have predominantly been designed around patient-level endpoints and less frequently around staff workloads, resource utilization, waiting times, or costs. This imbalance is particularly relevant from a health services perspective, because the scalability and sustainability of AI depend ultimately on whether clinical benefits can be achieved without generating disproportionate implementation costs or organizational burdens. For instance, a recent systematic review by Wenderott et al. found that, while 67% of studies on AI in medical imaging reported reduced processing times, meta-analyses did not confirm significant effects, and substantial heterogeneity persisted, illustrating the complexity of translating apparent efficiency gains into robust conclusions [85]. The geographical concentration of evidence in the USA and East Asian settings further limits conclusions regarding transferability to European and publicly funded healthcare systems, where the organizational structures and care pathways may differ substantially. Differences in effect magnitude across regions should not be interpreted as direct evidence of publication bias but as a signal of uncertainty regarding external validity.

**Summary of Key Findings**

- **Overall signal:** Overall, 74.1% of the included RCTs (40/54) reported a positive direction of effect on their primary endpoint, with heterogeneous effect sizes and variable certainty of evidence.
- **Perceptive AI:** Consistent positive results in diagnostic accuracy (colonoscopy CADe, ECG-based screening) were reported; alert fatigue limits clinical impact when outputs are not workflow-integrated.
- **Predictive/decision support AI:** Effectiveness critically depends on structural integration into clinical workflows; passive alert delivery was associated with limited uptake (22–30%) and a minimal impact on outcomes.
- **Conversational/Adaptive AI:** This yielded the most consistent positive signal, particularly for chronic disease management, behavioral support, and therapeutic adherence; it should be interpreted with caution given the high RoB in this subgroup (6/22 studies with high risk).
- **Organizational outcomes:** These are substantially underrepresented (three studies were exclusively organizational); there is insufficient evidence to draw conclusions on cost-effectiveness or scalability.
- **Generalizability:** Evidence is concentrated in the USA (*n* = 17) and China (*n* = 8); there are limited data from low- and middle-income healthcare systems.
- **Research priorities:** Pragmatic multicenter RCTs with standardized outcome reporting, longer follow-up, and explicit organizational and implementation outcomes are needed.

### 4.4. Limitations

Several limitations should be acknowledged. The substantial heterogeneity of the included interventions, populations, and outcomes precluded meta-analytic pooling, limiting the conclusions to a structured narrative synthesis. Given that a formal sensitivity analysis requires pooled effect estimates, it could not be performed in the conventional sense within a narrative synthesis framework. Nevertheless, the potential influence of studies rated as having a high risk of bias was addressed narratively: within each functional AI subgroup, the direction-of-effect findings are discussed separately for high-risk studies and low-risk/some-concerns studies in Section 3.5.1, Section 3.5.2 and Section 3.5.3. Readers should note that high-risk studies are concentrated in the conversational/adaptive AI subgroup (6 of 22 studies), where positive findings should be interpreted with particular caution given the high rates of open-label designs, self-reported outcomes, and short follow-up periods. Despite restricting inclusion to RCTs, several studies exhibited methodological concerns, including small samples, short follow-up periods, and challenges in blinding participants to AI-based interventions—an inherent rather than avoidable limitation in this field. The post hoc functional taxonomy, while grounded in established international frameworks, introduces a degree of classification subjectivity; this was mitigated by independent dual-reviewer classification with arbitration. The review included only English-language publications, which may have introduced language bias. The geographical concentration of evidence in the USA (*n* = 17) and China (*n* = 8) limits conclusions regarding transferability to other healthcare systems, particularly those in low- and middle-income countries. Finally, given the rapid pace of AI development, some interventions evaluated in earlier studies may not reflect the current state of the technology, and findings from studies published up to January 2026 may not capture the most recent generative AI applications in healthcare.

The heterogeneity of the included studies extends beyond the AI technology type to encompass the clinical context, patient population, healthcare system, comparator design, and outcome measurement, making it difficult to isolate the contribution of any single factor to the observed direction of effect. In particular, comparator choices varied from no intervention to active human-delivered treatment, which represents a substantively different benchmark and limits the comparability of effect estimates across studies. The predominance of short follow-up periods (median of approximately 12 weeks across all included studies) means that the maintenance of effects, long-term safety, and the sustainability of implementation gains remain unknown.

## 5. Conclusions

This systematic review of 54 randomized controlled trials indicates that AI-based interventions are associated with a predominantly positive direction of effect in real-world healthcare settings, with 40 of 54 studies (74.1%) reporting a benefit for their primary endpoint. However, the certainty, clinical relevance, and generalizability of these findings vary substantially across functional AI categories, implementation contexts, and outcome domains. Statistically significant results were more common than formally adjudicated clinical importance, and several positive classifications were based on intermediate, process, or engagement outcomes rather than on patient-level health endpoints. Conversational/adaptive AI showed the most consistent positive signal, particularly for treatment adherence, chronic disease self-management, and behavioral support, whereas predictive/decision support AI showed greater variability, with its effectiveness contingent on structural integration into clinical workflows. These findings suggest that the successful implementation of AI in healthcare should be guided not only by technical performance but also by workflow compatibility, clinician and patient engagement, and the rigorous evaluation of both clinical and organizational outcomes. Future research should prioritize pragmatic multicenter trials with longer follow-up, standardized outcome reporting, and the explicit evaluation of implementation costs and sustainability, particularly in healthcare systems that remain underrepresented in the current evidence base.

## Figures and Tables

**Table 1:** Characteristics and results of included studies. Color coding for direction of effect: green = positive; yellow = neutral; blue = mixed; red = negative. Citation numbers in brackets correspond to the reference list.

Author and Year	Country	Study Design	Population	AI Type	AI Function	Comparator	Primary Outcome	Key Result	Direction	Domain
Aguilera et al., 2024 [23]	USA	RCT	*N* = 168 adults with diabetes and depression; English/Spanish language preference; 57 control, 56 random messaging, 55 adaptive messaging	Conversational/adaptive AI	Treatment/monitoring	Control app (non-adaptive messaging)	Change in daily step count at 24 weeks	Adaptive messaging: +19% steps vs. control +3.9% vs. random messaging +1.6%	Positive	Clinical
Ahmad et al., 2023 [24]	UK	RCT	*N* = 614 patients aged 60–74 years with a positive fecal test and 55-year-olds with large adenomas, randomized 1:1 to CADe-assisted vs. standard colonoscopy	Perceptive AI	Diagnosis	Standard colonoscopy (no CADe)	Polyp detection rate (PDR)	PDR 85.7% vs. 79.7% (*p* = 0.05); ADR 71.4% vs. 65.0% (*p* = 0.09); significant polyp detection rate 79.2% vs. 71.6% (*p* = 0.03)	Mixed	Clinical
Alfonsi et al., 2020 [25]	Canada	Pilot RCT	*N* = 44 youths aged 10–17 years with type 1 diabetes for at least 6 months; 22 iSpy, 22 usual care	Perceptive AI	Education/training	Standard carbohydrate counting (no app)	Change in carbohydrate-counting ability at 3 months	Better carbohydrate-counting accuracy (*p* = 0.008), fewer large errors (*p* = 0.047), lower HbA1c (*p* = 0.03)	Positive	Clinical
Anan et al., 2021 [26]	Japan	RCT	*N* = 121 sedentary office workers and engineers (aged 20–64 years) suffering from neck/shoulder stiffness and low back pain	Conversational/adaptive AI	Treatment/monitoring	Control (no intervention)	Subjective pain severity and improvement	Symptom improvement OR: 43.00 (*p* < 0.001); adherence rate 92%	Positive	Clinical
Blomberg et al., 2021 [27]	Denmark	RCT	*N* = 5242 emergency calls received by the 112 medical dispatch center evaluated by the machine learning model	Perceptive AI	Diagnosis	Standard dispatcher protocol	Cardiac arrest recognition rate by dispatchers	OHCA recognition rate: 93.7% vs. 90.5% (*p* = 0.15); AI detected more cases, but human operators often ignored the alerts, negating the potential benefit	Neutral	Clinical
Carrasco-Hernandez et al., 2020 [28]	Spain	RCT	*N* = 240 adult smokers; 120 intervention, 120 control; recruited in an outpatient smoking cessation unit	Conversational/adaptive AI	Treatment/monitoring	Standard smoking cessation counselling	Smoking abstinence at 1 year	Abstinence 64.7% vs. 40.0% per-protocol and 27.5% vs. 15.0% intention-to-treat (*p* ≤ 0.02)	Positive	Clinical
Dean et al., 2022 [29]	USA	RCT	*N* = 6848 adult patients (median age 67 years) arriving at the emergency department with confirmed pneumonia diagnosis	Predictive/decision support AI	CDSS	Standard clinical care (no CDSS)	Severity-adjusted 30-day all-cause mortality	30-day all-cause mortality aOR: 0.62 (*p* < 0.001); 17% increase in direct home discharge	Positive	Mixed
Desai et al., 2023 [30]	USA	Multicenter, prospective RCT	*N* = 1033 average-risk subjects (age 45+) undergoing screening/surveillance colonoscopy	Perceptive AI	Diagnosis	Standard colonoscopy (no AI)	Adenomas per colonoscopy (APC) and positive predictive value (PPV)	APC: 0.99 ± 1.6 (CAC) vs. 0.85 ± 1.5 (CC), *p* = 0.02, IRR 1.17 [1.03–1.33]	Positive	Clinical
Doherty et al., 2024 [31]	Ireland	RCT	*N* = 62 healthy adults (24 men, 38 women; mean age 43 years) from Dublin; app-based exercise intervention	Conversational/adaptive AI	Treatment/monitoring	Standard exercise app (fixed prescription)	User satisfaction (PACES-8)	Satisfaction 4.0 (3.9–4.1) vs. 3.73 (3.6–3.8), *p* = 0.02; intensity 5.82 (5.59–6.05) vs. 5.19 (4.97–5.41), *p* < 0.01	Positive	Clinical
Fazlollahi et al., 2022 [32]	Canada	RCT	*N* = 70 Medical students (mean age 21.8 years) with no prior surgical experience	Predictive/decision support AI	Education/training	Remote expert instruction	Surgical performance improvement over time and skill retention	Expertise score improvement: +0.66 points (*p* < 0.001); AI tutoring is significantly better than remote human experts for training surgery students	Positive	Organizational
Fitzpatrick et al., 2017 [33]	USA	RCT	*N* = 70 college students aged 18–28 years with anxiety and depression symptoms	Conversational/adaptive AI	Treatment/monitoring	Waitlist control	Depressive symptoms (PHQ-9)	Significant reduction in depression (*p* = 0.01) and anxiety (*p* = 0.004); mean interactions 12.14	Positive	Clinical
Fritz et al., 2024 [34]	USA	RCT (single-center, prospective)	*N* = 5071 surgical patients (mean age 58 years, 52% female); 89 clinicians	Predictive/decision support AI	Clinical Decision Support (CDSS)	Unassisted clinician prediction	AUROC of clinician predictions for 30-day mortality and AKI (ML-assisted vs. unassisted)	AUROC mortality: 0.793 vs. 0.780, diff. 0.013 [95% CI−0.070–0.097], *p* = 0.76; AUROC AKI: 0.734 vs. 0.688, *p* = 0.06	Neutral	Clinical
Fulmer et al., 2018 [35]	USA	RCT	*N* = 75 US college participants with depression/anxiety symptoms; Tess 2-week group *n* = 24, 4-week group *n* = 26, control *n* = 24	Conversational/adaptive AI	Treatment/monitoring	Waitlist control	Depressive/anxiety symptoms (PHQ-9, GAD-7)	Reduction in depression PHQ-9 (*p* = 0.02); anxiety GAD-7 group 1 *p* = 0.045 and group 2 *p* = 0.02; PANAS difference *p* = 0.03 vs. control	Positive	Clinical
Gong et al., 2020 [36]	China	RCT	*N* = 355 ENDOANGEL, 349 control	Perceptive AI	Diagnosis	Standard colonoscopy (no ENDOANGEL)	Adenoma detection rate (ADR)	ADR: 16% vs. 8% (ITT), *p* = 0.0010	Positive	Clinical
Halcox et al., 2017 [37]	UK	RCT	*N* = 1000 patients ≥ 65 years, CHADS-VASc ≥ 2, without atrial fibrillation; 499 IECG, 501 routine care	Perceptive AI	Diagnosis	Routine clinical care (no AliveCor)	Time to diagnosis of atrial fibrillation	Higher AF diagnosis rate with IECG: HR = 3.9, 95% CI 1.4–10.4, *p* = 0.007; cost per diagnosis $10,780 (£8255)	Positive	Mixed
Harry et al., 2022 [38]	USA	RCT	*N* = 5054 young adults (aged 18–26 years) due for the human papillomavirus (HPV) vaccination	Predictive/decision support AI	CDSS	Standard EHR care (no CDSS alert)	HPV vaccination series completion and receipt of ≥1 HPV vaccine dose during the study period	HPV vaccination series completion: 2.3% vs. 2.2% (*p* = 0.827); no significant difference between groups	Neutral	Mixed
He et al., 2024 [39]	China	RCT	*N* = 75 older adults (aged 60–75 years) with sarcopenia recruited from community organizations	Perceptive AI	Treatment/monitoring	In-person rehabilitation program	Muscular and functional outcomes (ASMI, grip strength, TUGT) and quality of life (QoL)	ASMI diff: −0.011 kg/m2 (*p* > 0.05); AI is as effective as in-person traditional training, but not clinically superior	Neutral	Clinical
Chen et al., 2023 [40]	Taiwan	RCT	*N* = 94 participants aged 20–65 years with schizophrenia in psychiatric day centers; 59 control, 35 experimental	Perceptive AI	Treatment/monitoring	Routine clinical care (no AI monitoring)	Medication adherence rate	Adherence 94.72% vs. 64.43%; significant improvement in symptoms and cognition (*p* < 0.001)	Positive	Clinical
Itoh et al., 2022 [41]	Japan	RCT	*N* = 101 workers (mean age ~47 years) suffering from chronic low back pain and undergoing pharmacological treatment	Predictive/decision support AI	Treatment/monitoring	Control group (no mobile program)	Work productivity change at 12 weeks	Work productivity diff (QQ): −0.053 (*p* = 0.43); did not improve work productivity but effectively reduced back pain and fear of movement	Neutral	Mixed
Jayakumar et al., 2021 [42]	USA	RCT	*N* = 129 adults with knee osteoarthritis (69 intervention, 60 control)	Predictive/decision support AI	Clinical Decision Support (CDSS)	Educational brochure (standard information)	Decision quality (K-DQI)	K-DQI: mean difference 20.0% [95% CI 14.2–26.1%], *p* < 0.001; better SDM, satisfaction, and function at 4–6 months	Positive	Clinical
Kanazawa et al., 2023 [43]	Japan	RCT with crossover design	*N* = 20 resident physicians conducting mock medical interviews with 10 simulated patients	Predictive/decision support AI	CDSS	Standard interview without AI assistance	Diagnostic accuracy (correct answer rate for the top 3 differential diagnoses listed)	Diagnostic accuracy: 56.1% vs. 39.3% (*p* = 0.02); AI helps resident physicians to diagnose more accurately and conduct slightly faster interviews	Positive	Mixed
Knitza et al., 2024 [44]	Germany	RCT	*N* = 600 patients (median age 52 years, ~70% female) awaiting consultation at rheumatology centers	Predictive/decision support AI	Diagnosis	Physician clinical diagnosis	Concordant detection of any inflammatory rheumatic disease (IRD) between AI tool and physician diagnosis	Diagnostic accuracy: Ada 58%, Rheport 52%; both tools performed poorly as standalone diagnostic triage and should not replace physicians	Negative	Clinical
Lee, Byun et al., 2025 [45]	Republic of Korea	RCT (4 arms)	*N* = 145 breast cancer patients undergoing postoperative radiation therapy	Conversational/adaptive AI	Education/training	Routine patient education (no chatbot)	Anxiety measured via APAIS (primary); STAI and LASA (secondary)	No significant group × time interaction for APAIS, STAI, or LASA; trend only in unplanned subgroup analysis	Neutral	Clinical
Lee et al., 2023 [46]	Republic of Korea	Open-label RCT, 3 arms	*N* = 294 patients with type 2 diabetes; 3 university hospitals; 48 weeks	Conversational/adaptive AI	Treatment/monitoring	Standard care	Change in HbA1c at 48 weeks	HbA1c significantly lower in platform (B) and platform + CGM + feedback (C) vs. routine care (A)	Positive	Clinical
Li et al., 2025 [47]	China	RCT	*N* = 47 post-surgery NSCLC survivors; 40 completed the trial; home-based cardiopulmonary rehabilitation	Conversational/adaptive AI	Treatment/monitoring	Standard care	Cardiorespiratory capacity (VO2 peak)	VO2 peak +3.66 vs. +1.09 mL/kg/min (*p* = 0.02); QOL 16.25 vs. 1.04 (*p* = 0.03); reduced fatigue and anxiety	Positive	Clinical
Li et al., 2024 [48]	China	RCT	*N* = 100 adult patients with stage II/III periodontitis	Perceptive AI	Treatment/monitoring	Standard care	Resolution of inflamed periodontal pockets	Inflamed pocket diff: 7.9% reduction (*p* = 0.015); multimodal AI effectively improves real-world dental hygiene and disease healing	Positive	Mixed
Lin et al., 2024 [49]	Taiwan	Pragmatic, multisite RCT	*N* = 15,965 hospitalized patients; 39 physicians	Perceptive AI	Prediction	Standard ECG interpretation (no AI alert)	All-cause mortality at 90 days	3.6% (intervention) vs. 4.3% (control), HR 0.83 [95% CI 0.70–0.99]; high-risk subgroup: HR 0.69 [0.53–0.90]	Positive	Clinical
Lustrek et al., 2021 [50]	Belgium/Italy	RCT	*N* = 56 outpatients with stable heart failure, anxiety, and depression	Conversational/adaptive AI	Treatment/monitoring	Standard heart failure follow-up	Self-management/self-care in heart failure	Improved self-care, reduced depression/anxiety; systolic blood pressure measurement error 9.0 mmHg and diastolic 7.0 mmHg	Positive	Clinical
Ly et al., 2017 [51]	Sweden	Pilot RCT	*N* = 28 non-clinical participants; 14 Shim chatbot intervention, 14 waitlist	Conversational/adaptive AI	Treatment/monitoring	Waitlist control	Psychological well-being/stress	Improved well-being and reduced stress during the 2-week intervention; high engagement	Positive	Clinical
Manz et al., 2023 [52]	USA	RCT	*N* = 20,506 cancer outpatients at high or low risk for 180-day mortality, managed by oncology clinicians	Predictive/decision support AI	CDSS	Standard oncology care (no ML nudge)	Rate of documented Serious Illness Conversations (SICs) per 100 unique patient visits	Serious illness conv. rate: 3.4% to 13.5% (aOR: 2.62, *p* < 0.001	Positive	Mixed
Mathioudakis et al., 2025 [53]	USA	Pragmatic, phase 3 noninferiority RCT	*N* = 368 adults with prediabetes and overweight/obesity (median age 58 years, 71% female)	Conversational/adaptive AI	Treatment/monitoring	Human lifestyle coach (DPP standard)	Composite: HbA1c < 6.5% + ≥5% weight loss, or ≥4% weight loss + physical activity, or HbA1c reduction ≥ 0.2 at 12 months	31.7% (AI) vs. 31.9% (human coach), risk difference −0.2% [1-sided 95% CI −8.2%]; noninferiority met	Neutral	Clinical
Nayak et al., 2023 [54]	USA	RCT	*N* = 32 adults with type 2 diabetes requiring initiation or adjustment of basal insulin	Conversational/adaptive AI	Treatment/monitoring	Standard insulin titration by clinician	Time to achieving the optimal basal insulin dose	Time to optimal dose 15 days vs. >56; glycemic control 81.3% vs. 25.0%; better adherence and lower distress	Positive	Clinical
Nicolae et al., 2020 [55]	Canada	RCT	*N* = 41 patients with low/intermediate-risk prostate cancer treated with LDR I-125 brachytherapy; PIPA *n* = 21, control *n* = 20	Predictive/decision support AI	CDSS	Manual brachytherapy planning	Noninferiority of 30-day dosimetry compared with conventional planning	No dosimetric difference between PIPA and conventional planning; faster planning time with PIPA	Positive	Mixed
Olano-Espinosa et al., 2022 [56]	Spain	RCT	*N* = 513 adult smokers (18+); 242 chatbot, 271 usual care; recruited in 34 primary care centers	Conversational/adaptive AI	Treatment/monitoring	Standard primary care cessation support	Smoking abstinence at 6 months validated by CO-oximetry	Abstinence 26.0% vs. 18.8%; *p* = 0.05	Positive	Clinical
Pelle et al., 2020 [57]	The Netherlands	Open-label RCT	*N* = 427 participants aged 50 years and older suffering from osteoarthritis of the knee and/or hip	Conversational/adaptive AI	Treatment/monitoring	Usual care (no app)	Number of secondary healthcare consultations (orthopedic surgeon, rheumatologist, physiotherapist, general practitioner)	Secondary healthcare consultations IRR: 1.20 (*p* > 0.05); small improvements in pain (+3.5 points) and symptoms (+2.6 points) on 0–100 scale	Neutral	Mixed
Piette et al., 2022 [58]	USA	RCT	*N* = 439 adults with chronic back pain (mean age 53.6 years; 40.5% female; 31.0% Black/African American)	Conversational/adaptive AI	Treatment/monitoring	Telephone-delivered CBT	Pain-related disability (Roland-Morris Disability Questionnaire, RMDQ) and pain intensity (Brief Pain Inventory, BPI)	Significant reductions in pain disability (RMDQ) and intensity (BPI) at 3, 6, and 12 months in both AI and telephone CBT arms; AI non-inferior to telephone CBT at ∼50% lower therapist time	Positive	Mixed
Prochaska et al., 2021 [59]	USA	RCT	*N* = 180 US adults, mean age 40 years, 65% women, positive screening for substance misuse; 88 Woebot-SUDs, 92 waitlist	Conversational/adaptive AI	Treatment/monitoring	Control (psychoeducation only)	Change in occasions of substance use in the past month (baseline–EOT)	Reduction of 9.1 occasions vs. 3.3 in the waitlist (*p* = 0.039)	Positive	Clinical
Repici et al., 2020 [60]	Italy	RCT	*N* = 685 adults aged 40–80 years undergoing colonoscopy for screening, surveillance, or symptoms; 341 CADe, 344 control	Perceptive AI	Diagnosis	Standard colonoscopy (no CADe)	Adenoma detection rate (ADR)	ADR 54.8% vs. 40.4%; RR 1.30. APC 1.07 ± 1.54 vs. 0.71 ± 1.20; IRR 1.46	Positive	Clinical
Sadeh-Sharvit et al., 2023 [61]	USA	RCT	*N* = 47 adults with depressive and/or anxiety disorders	Predictive/decision support AI	CDSS	Standard therapist-led care	Feasibility and acceptability of the platform	Note submission time: 14 h vs. 69 h (*p* < 0.001); no significant difference in clinical outcomes between groups	Positive	Mixed
Seol et al., 2021 [62]	USA	RCT	*N* = 184 children (mean age 9.0 years) with persistent asthma or high risk for exacerbations, managed by primary care clinicians	Predictive/decision support AI	CDSS	Standard asthma management (no CDSS)	Asthma exacerbations and decision time	EHR review time: 3.5 vs. 11.3 min (*p* < 0.001); asthma exacerbation rate: 12% vs. 15% (*p* = NS)	Mixed	Mixed
Shade et al., 2025 [63]	USA	RCT	*N* = 50 older adults (aged 60 and over) who live alone and suffer from chronic musculoskeletal pain	Conversational/adaptive AI	Treatment/monitoring	Standard voice assistant (non-personalized)	AI routine engagement and system usability	Morning routine initiations: 74% vs. 58% (*p* = 0.002); AI voice assistants were associated with higher engagement rates in older adults with chronic pain	Positive	Mixed
Sharma et al., 2023 [64]	Canada	RCT with crossover	*N* = 52 participants (heart failure patients and caregivers), 69% male, median age 51 years	Conversational/adaptive AI	Diagnosis	Standard care	Concordance between AI voice assistant and healthcare staff (% agreement and kappa)	Concordance 96.9–98.5%, kappa 0.92–0.95 [95% CI 0.84–1.00]; no statistical difference between groups (*p* > 0.05)	Neutral	Clinical
Sharma et al., 2025 [65]	UK	Cluster RCT + diagnostic accuracy study	*N* = 396 adults requiring referral for retinal disease (AI data for 204 participants)	Perceptive AI	Clinical Decision Support (CDSS)	Standard care	Proportion of false-positive urgent referrals; sensitivity/specificity of AI model	Tele-ophthalmology reduced false-positive urgent referrals by 59% vs. standard care; AI model showed lower sensitivity/specificity than human experts	Mixed	Both
Sharp et al., 2025 [66]	Australia	Two-arm RCT	*N* = 60 (30 intervention, 30 control) on waitlist for eating disorder treatment	Conversational/adaptive AI	Treatment/monitoring	Waitlist control (no chatbot)	Global EDE-Q score at 1 and 3 months	Significant group × time effect (F2,116=5.98, *p* = 0.003, ηp^2^ = 0.09); in-person treatment initiation: 93% (chatbot) vs. 70% (control), *p* = 0.042	Positive	Clinical
Sibley et al., 2025 [67]	USA	RCT	*N* = 51 adolescents with ADHD; 23 therapists across 3 community mental health agencies	Predictive/decision support AI	Clinical Decision Support (CDSS)	Standard implementation support	Treatment delivery efficiency (appointments attended) and implementation quality (fidelity, MI quality)	AI-assisted group: more efficient treatment delivery but fewer appointments attended; significant decline in MI quality over time	Mixed	Organizational
Su et al., 2020 [68]	China	RCT	*N* = 659 patients undergoing routine colonoscopy for colorectal cancer screening/diagnosis; 308 intervention, 315 control	Perceptive AI	Diagnosis	Standard colonoscopy (sham CADe)	Adenoma detection rate (ADR)	ADR 28.9% vs. 16.5%; PDR 38.3% vs. 25.4%; adenomas/procedure 0.367 vs. 0.178; withdrawal time 7.03 vs. 5.81 min (all *p* < 0.001)	Positive	Mixed
Tawfik et al., 2023 [69]	Egypt	3-arm RCT	*N* = 150 breast cancer patients undergoing chemotherapy (50 per arm)	Conversational/adaptive AI	Education/training	Routine chemotherapy care	Symptom frequency/severity/distress; self-care behaviors	Significant improvement in patient-centered outcomes in chatbot and nurse-led groups vs. routine care	Positive	Clinical
Turino et al., 2021 [70]	Spain	RCT	*N* = 60 newly diagnosed patients with severe obstructive sleep apnea (OSA) requiring CPAP therapy	Conversational/adaptive AI	Treatment/monitoring	Standard CPAP follow-up	CPAP adherence at 6 months	CPAP compliance diff: +1.14 h/day (*p* = 0.047); AI-assisted remote monitoring was associated with improved therapy adherence without additional costs	Positive	Mixed
Wallace et al., 2022 [71]	Italy, UK, USA	Tandem RCT (8 centers)	*N* = 230 subjects undergoing CRC screening/surveillance with same-day back-to-back colonoscopies	Perceptive AI	Diagnosis	Standard colonoscopy (tandem, AI-second)	Adenoma miss rate (AMR)	AMR 15.5% (AI-first) vs. 32.4% (non-AI-first), adjusted OR 0.38 [95% CI 0.23–0.62]; false-negative rate 6.8% vs. 29.6%	Positive	Clinical
Wang et al., 2020 [72]	China	RCT	*N* = 962 patients aged 18–75 years; 484 CADe, 478 sham colonoscopy	Perceptive AI	Diagnosis	Standard colonoscopy (sham CADe)	Adenoma detection rate (ADR)	CADe-assisted colonoscopy significantly reduced adenoma miss rate (13.89% vs. 40.00%, *p* < 0.0001) and polyp miss rate (13.54% vs. 39.58%, *p* < 0.0001)	Mixed	Clinical
Wijnberge et al., 2020 [73]	The Netherlands	RCT	*N* = 68 adult patients (median age 64 years) undergoing elective noncardiac surgery under general anesthesia	Predictive/decision support AI	Prediction	Standard anesthesia management	Time-weighted average of intraoperative hypotension	Hypotension time-weighted avg: 0.10 vs. 0.44 mmHg (*p* = 0.001); the intervention was associated with significantly reduced intraoperative hypotension	Positive	Mixed
Wright et al., 2023 [74]	USA	RCT	*N* = 288,832 clinical opportunities (alerts analyzed) among providers at multi-site academic medical centers	Predictive/decision support AI	CDSS	Standard EHR alerts (no AI-CDSS)	Problem list completeness (alert acceptance rate and number of missing clinical diagnoses added per encounter)	Missing diagnoses added to EHR: 4.6 times more than standard care (*p* < 0.0001); alert acceptance rate 22.1%	Positive	Organizational
Xiao et al., 2025 [75]	China	Open-label, double-arm RCT	*N* = 38 randomized (34 analyzed) with chronic nonspecific low back pain	Perceptive AI	Treatment/monitoring	Video-based exercise program	Change in most severe NRS pain score at week 4	Most severe NRS: −3.00 (AI) vs. −1.50 (video), adjusted mean difference −1.08 [95% CI −1.68 to −0.49], *p* < 0.001	Positive	Clinical
Yao et al., 2021 [76]	USA	Pragmatic, cluster-randomized clinical trial	*N* = 22,641 adults without prior heart failure; 120 primary care teams, 358 clinicians	Perceptive AI	Diagnosis	Standard ECG interpretation (no AI alert)	New diagnosis of low ejection fraction (≤50%) within 90 days of the ECG	2.1% (intervention) vs. 1.6% (control), OR 1.32 [1.01–1.61], *p* = 0.007; AI-ECG-positive subgroup: OR 1.43 [1.08–1.91]	Positive	Clinical

ADR = adenoma detection rate; PDR = polyp detection rate; CADe = computer-aided detection; CDSS = clinical decision support system; RR = risk ratio; HR = hazard ratio; aOR = adjusted odds ratio; IRR = incidence rate ratio. Citation numbers in brackets correspond to the reference list.

**Table 2:** Distribution of included studies (*n* = 54) by AI functional category and primary outcome domain. Clinical = studies reporting exclusively patient-level clinical outcomes; organizational = studies reporting exclusively workflow, process, or resource use outcomes; mixed = studies reporting both clinical and organizational outcomes.

AI Functional Category	Clinical Outcomes	Organizational Outcomes	Mixed Outcomes	Total
Perceptive AI	14	0	3	17
Predictive/decision support AI	9	2	4	15
Conversational/adaptive AI	11	1	10	22
Total	34	3	17	54

## Data Availability

No new data were created or analyzed in this study. Data sharing is not applicable to this article.

## Supplementary Materials

The following supporting information can be downloaded at https://www.mdpi.com/article/10.3390/healthcare14203532/s1. Supplementary Material S1: Complete Database Search Strategies.
