# Supplementary material for: Effectiveness of Artificial Intelligence Systems in Healthcare: A Systematic Review of Clinical and Organizational Outcomes
Source: Healthcare (Basel). 2026 Oct 20;14(20):3532. doi: 10.3390/healthcare14203532 (PMC13704068; doi:10.3390/healthcare14203532)
Supplement: Supplementary file 1 [file healthcare-14-03532-s001.zip › healthcare-4542457-supplementary.pdf]

# Supplementary Material S1

## Complete Database Search Strategies

*Effectiveness of Artificial Intelligence Systems in Healthcare: A Systematic Review of Clinical and Organizational Outcomes*

## Overview of Database Searches

The systematic review was conducted across four databases. The following table summarises the search parameters for each database. Complete Boolean search strings are provided in the subsequent sections.

| Database | Platform | Records Retrieved | Filters Applied |
|---|---|---|---|
| MEDLINE (PubMed) | pubmed.ncbi.nlm.nih.gov | 2,044 | 2015–2026; English; Primary studies |
| CINAHL | EBSCOhost | 505 | 2015–2026; English; Peer-reviewed |
| Cochrane CENTRAL | Cochrane Library (cochranelibrary.com) | 1,093 | 2015–2026; English |
| ClinicalTrials.gov | clinicaltrials.gov | 2,119 | Completed trials with results; 2015–2026 |
| **Total** 5,761 records retrieved before deduplication | | | |

## S1.1 MEDLINE (PubMed)

Platform: PubMed (pubmed.ncbi.nlm.nih.gov) | Search date: January 2026 | Records retrieved: 2,044

Filters: Publication date 01/01/2015–01/01/2026; English language; Primary studies only (reviews, meta-analyses, protocols excluded)

**Search String:**

```
("Artificial Intelligence"[MeSH] OR "Machine Learning"[MeSH] OR "Deep
Learning"[MeSH] OR "Neural Networks, Computer"[MeSH] OR "Natural Language
Processing"[MeSH] OR "Decision Support Systems, Clinical"[MeSH] OR "Artificial
Intelligence"[TIAB] OR "Machine Learning"[TIAB] OR "Deep Learning"[TIAB] OR "Neural
Networks"[TIAB] OR "Natural Language Processing"[TIAB] OR "AI-driven"[TIAB] OR
"Algorithm-based"[TIAB])  AND  ("Healthcare"[MeSH] OR "Health System"[TIAB] OR
"Healthcare System"[TIAB] OR "Health Service*"[TIAB] OR "Healthcare Delivery"[TIAB]
OR "Healthcare Organization*"[TIAB] OR "Healthcare Infrastructure*"[TIAB] OR
"Health Services Delivery"[TIAB] OR "Health Care Delivery"[TIAB] OR "Health Care
System"[TIAB] OR "Public Health System*"[TIAB] OR "Medical System*"[TIAB] OR
"Health Management"[TIAB] OR "Medical Care"[TIAB] OR "Clinical Care"[TIAB] OR
"Healthcare Provision"[TIAB] OR "Health Provision"[TIAB] OR "Medical
Services"[TIAB] OR "Health Support*"[TIAB] OR "Health Services Provision"[TIAB] OR
"Telemedicine"[MeSH] OR "Telehealth"[TIAB] OR "eHealth"[TIAB] OR "Digital
Health"[TIAB] OR "Hospital Management"[TIAB] OR "Patient Care"[TIAB] OR "Clinical
Workflow"[TIAB])  AND  ("Effectiveness"[TIAB] OR "Efficacy"[TIAB] OR
"Outcome*"[TIAB] OR "Clinical Outcome*"[TIAB] OR "Health Outcome*"[TIAB] OR
"Treatment Outcome*"[TIAB] OR "Impact*"[TIAB] OR "Evaluation*"[TIAB] OR
"Performance*"[TIAB] OR "Assessment"[TIAB] OR "Cost-effectiveness"[TIAB] OR "Impact
Assessment"[TIAB] OR "Efficiency"[TIAB] OR "Validation"[TIAB] OR "Outcome
Assessment*"[TIAB] OR "Predictive Value"[TIAB] OR "Diagnostic Accuracy"[TIAB] OR
```

"Clinical Decision-Making"[TIAB] OR "Risk Stratification*"[TIAB] OR "Quality of Care"[TIAB] OR "Patient Safety"[TIAB] OR "Medical Errors"[TIAB])

## S1.2 CINAHL (EBSCOhost)

Platform: EBSCOhost | Search date: January 2026 | Records retrieved: 505

Filters applied (not part of Boolean string): full-text availability; peer-reviewed journals; academic publications; publication date 01/01/2015–01/01/2026; English language

**Search String:**

```
(MH "Artificial Intelligence+" OR MH "Machine Learning+" OR MH "Deep Learning" OR
MH "Neural Networks+" OR MH "Natural Language Processing+" OR MH "Decision Support
Systems, Clinical" OR TI ("Artificial Intelligence" OR "Machine Learning" OR "Deep
Learning" OR "Neural Networks" OR "Natural Language Processing" OR "AI-driven" OR
"Algorithm-based") OR AB ("Artificial Intelligence" OR "Machine Learning" OR "Deep
Learning" OR "Neural Networks" OR "Natural Language Processing" OR "AI-driven" OR
"Algorithm-based"))  AND  (MH "Health Services+" OR MH "Health Care Delivery+" OR
MH "Patient Care+" OR MH "Telemedicine+" OR TI ("Health System*" OR "Healthcare
System*" OR "Health Service*" OR "Healthcare Delivery" OR "Healthcare
Organization*" OR "Healthcare Infrastructure*" OR "Health Services Delivery" OR
"Health Care Delivery" OR "Health Care System*" OR "Public Health System*" OR
"Medical System*" OR "Health Management" OR "Medical Care" OR "Clinical Care" OR
"Healthcare Provision" OR "Health Provision" OR "Medical Services" OR "Health
Support*" OR "Health Services Provision" OR "Telemedicine" OR "Telehealth" OR
"eHealth" OR "Digital Health" OR "Hospital Management" OR "Patient Care" OR
"Clinical Workflow") OR AB ("Health System*" OR "Healthcare System*" OR "Health
Service*" OR "Healthcare Delivery" OR "Healthcare Organization*" OR "Healthcare
Infrastructure*" OR "Health Services Delivery" OR "Health Care Delivery" OR "Health
Care System*" OR "Public Health System*" OR "Medical System*" OR "Health
Management" OR "Medical Care" OR "Clinical Care" OR "Healthcare Provision" OR
"Health Provision" OR "Medical Services" OR "Health Support*" OR "Health Services
Provision" OR "Telemedicine" OR "Telehealth" OR "eHealth" OR "Digital Health" OR
"Hospital Management" OR "Patient Care" OR "Clinical Workflow"))  AND  (TI
(Effectiveness OR Efficacy OR Outcome* OR "Clinical Outcome*" OR "Health Outcome*"
OR "Treatment Outcome*" OR Impact* OR Evaluation* OR Performance* OR Assessment OR
"Cost-effectiveness" OR "Impact Assessment" OR Efficiency OR Validation OR "Outcome
Assessment*" OR "Predictive Value" OR "Diagnostic Accuracy" OR "Clinical Decision-
Making" OR "Risk Stratification*" OR "Quality of Care" OR "Patient Safety" OR
"Medical Errors") OR AB (Effectiveness OR Efficacy OR Outcome* OR "Clinical
Outcome*" OR "Health Outcome*" OR "Treatment Outcome*" OR Impact* OR Evaluation* OR
Performance* OR Assessment OR "Cost-effectiveness" OR "Impact Assessment" OR
Efficiency OR Validation OR "Outcome Assessment*" OR "Predictive Value" OR
"Diagnostic Accuracy" OR "Clinical Decision-Making" OR "Risk Stratification*" OR
"Quality of Care" OR "Patient Safety" OR "Medical Errors"))
```

## S1.3 Cochrane CENTRAL

Platform: Cochrane Library (cochranelibrary.com) | Records retrieved: 1,093

Filters: Publication date 2015–2026; English language

**Search String:**

```
([mh "Artificial Intelligence"] OR [mh "Machine Learning"] OR [mh "Deep Learning"]
OR [mh "Neural Networks, Computer"] OR [mh "Natural Language Processing"] OR [mh
"Decision Support Systems, Clinical"] OR "Artificial Intelligence":ti,ab,kw OR
"Machine Learning":ti,ab,kw OR "Deep Learning":ti,ab,kw OR "Neural
Networks":ti,ab,kw OR "Natural Language Processing":ti,ab,kw OR "AI-
driven":ti,ab,kw OR "Algorithm-based":ti,ab,kw)  AND  ([mh "Healthcare"] OR [mh
"Telemedicine"] OR "Health System":ti,ab,kw OR "Healthcare System":ti,ab,kw OR
(Health NEXT Service*):ti,ab,kw OR "Healthcare Delivery":ti,ab,kw OR (Healthcare
NEXT Organization*):ti,ab,kw OR (Healthcare NEXT Infrastructure*):ti,ab,kw OR
"Health Services Delivery":ti,ab,kw OR "Health Care Delivery":ti,ab,kw OR "Health
Care System":ti,ab,kw OR (Public NEXT Health NEXT System*):ti,ab,kw OR (Medical
NEXT System*):ti,ab,kw OR "Health Management":ti,ab,kw OR "Medical Care":ti,ab,kw
OR "Clinical Care":ti,ab,kw OR "Healthcare Provision":ti,ab,kw OR "Health
Provision":ti,ab,kw OR "Medical Services":ti,ab,kw OR (Health NEXT
Support*):ti,ab,kw OR "Health Services Provision":ti,ab,kw OR "Telehealth":ti,ab,kw
OR "eHealth":ti,ab,kw OR "Digital Health":ti,ab,kw OR "Hospital
Management":ti,ab,kw OR "Patient Care":ti,ab,kw OR "Clinical Workflow":ti,ab,kw)
AND  ("Effectiveness":ti,ab,kw OR "Efficacy":ti,ab,kw OR Outcome*:ti,ab,kw OR
(Clinical NEXT Outcome*):ti,ab,kw OR (Health NEXT Outcome*):ti,ab,kw OR (Treatment
NEXT Outcome*):ti,ab,kw OR Impact*:ti,ab,kw OR Evaluation*:ti,ab,kw OR
Performance*:ti,ab,kw OR "Assessment":ti,ab,kw OR "Cost-effectiveness":ti,ab,kw OR
"Impact Assessment":ti,ab,kw OR "Efficiency":ti,ab,kw OR "Validation":ti,ab,kw OR
(Outcome NEXT Assessment*):ti,ab,kw OR "Predictive Value":ti,ab,kw OR "Diagnostic
Accuracy":ti,ab,kw OR "Clinical Decision-Making":ti,ab,kw OR (Risk NEXT
Stratification*):ti,ab,kw OR "Quality of Care":ti,ab,kw OR "Patient
Safety":ti,ab,kw OR "Medical Errors":ti,ab,kw)
```

## S1.4 ClinicalTrials.gov

Platform: clinicaltrials.gov | Search date: September 2026 | Records retrieved: 2,119

ClinicalTrials.gov was searched using the Advanced Search interface. Records were filtered to include only completed trials with posted results, conducted between 2015 and 2026. For each eligible trial registration, associated results publications were identified via the linked results section and cross-referenced with PubMed/CINAHL records to avoid duplication.

**Search String:**

```
("Artificial Intelligence" OR "Machine Learning" OR "Deep Learning" OR "Neural
Networks" OR "Natural Language Processing" OR "Decision Support Systems" OR "AI-
driven" OR "Algorithm-based")  AND  ("Healthcare" OR "Health System" OR "Healthcare
System" OR "Health Services" OR "Healthcare Delivery" OR "Healthcare Organization"
OR "Telemedicine" OR "Telehealth" OR "eHealth" OR "Digital Health" OR "Hospital
Management" OR "Patient Care" OR "Clinical Workflow")  AND  ("Effectiveness" OR
"Efficacy" OR "Outcomes" OR "Clinical Outcomes" OR "Health Outcomes" OR "Treatment
Outcomes" OR "Impact" OR "Evaluation" OR "Performance" OR "Assessment" OR "Cost-
effectiveness" OR "Efficiency" OR "Validation" OR "Predictive Value" OR "Diagnostic
Accuracy" OR "Decision Making" OR "Risk Stratification" OR "Quality of Care" OR
"Patient Safety" OR "Medical Errors")
```

## S1.5 Additional Notes on Search Methodology

### Deduplication

Duplicate records identified across databases were removed prior to screening. Records retrieved from ClinicalTrials.gov were handled as trial registrations; for each registration, associated results publications were identified and cross-referenced with PubMed and CINAHL records. Only registrations with posted results within the eligibility window were carried forward to full-text screening.

### PROSPERO Registration

This systematic review was registered on PROSPERO (CRD420251036790) prior to data extraction. The registered protocol specified PubMed, CINAHL, and ClinicalTrials.gov as primary databases. The addition of Cochrane CENTRAL and the extension of the search window from April 2025 to January 2026 represent pre-specified deviations from the registered protocol, undertaken to strengthen search comprehensiveness in response to peer review. These deviations are declared in Section 2.2 of the main manuscript. The PROSPERO record has been updated in line with the methodological changes made in agreement with the co-authors.
